# Outcome of Percutaneous Coronary Intervention in Relation to the Institutional Volume of Coronary Artery Bypass Surgery ^†^

**DOI:** 10.3390/jcm9051267

**Published:** 2020-04-27

**Authors:** Shun Kohsaka, Hiraku Kumamaru, Taku Inohara, Tetsuya Amano, Takashi Akasaka, Hiroaki Miyata, Noboru Motomura, Masato Nakamura

**Affiliations:** 1 Scientific and Registry Committee, Japanese Association of Cardiovascular Intervention and Therapeutics, Tokyo 104-0033, Japan; inohara-circ@umin.ac.jp (T.I.); amanot@aichi-med-u.ac.jp (T.A.); akasat@wakayama-med.ac.jp (T.A.); masato@oha.toho-u.ac.jp (M.N.); 2 Department of Healthcare Quality Assessment, Graduate School of Medicine, The University of Tokyo, Tokyo 113-8654, Japan; hik205@mail.harvard.edu (H.K.); h-m@keio.jp (H.M.); 3 Database Committee, Japan Cardiovascular Surgery Database, Tokyo 113-0033, Japan; noboru@motomura.org

**Keywords:** percutaneous coronary intervention, coronary artery bypass grafting, volume-outcome relationship, registry

## Abstract

Background: Percutaneous coronary intervention (PCI) is performed in a wide range of institutions. We sought to assess the relationship between coronary artery bypass grafting (CABG) volume relative to PCI volume and clinical outcome using nationally representative PCI and CABG registries in Japan. Methods: This was a collaborative, registry-based cohort study enrolling patients undergoing percutaneous coronary intervention in 2013–2014 using Japanese nationwide registry (J-PCI) with follow up until discharge. The absolute volume of CABG for each hospital was calculated using additional data from Japan CardioVascular Surgery Database (JCVSD). Patients undergoing their first PCI registered in the registry (N = 220,934), at 943 facilities were studied. Main outcomes were in-hospital mortality, and incidence of composite of in-hospital death and postprocedural complications. Results: Among the 220,934 patients, 162,411 were men, with a mean age of 69.7 (SD 11.6) years. Patients underwent PCI at hospitals with varying CABG volume: The overall in-hospital mortality and composite event rate for PCI patients was 0.9% and 2.4%, respectively. CABG volume was associated with the in-hospital mortality of PCI at facilities performing less than 200 PCIs per year, but not at facilities performing 200 or more. Similarly, in-hospital mortality or complication was associated with PCI volume <200 only if no CABG is done at the facility. The result remained largely consistent in subgroup of patients presenting with acute coronary syndrome or even after excluding these institutions with extremely low number of PCI (<50 cases/year) or CABG (<15 cases / year). Conclusions: In a nationwide registry-based analysis, the surgical volume was associated with patients’ clinical outcome after PCI, when limited number of PCIs were performed at the facility.

## 1. Introduction

Percutaneous coronary intervention (PCI) is performed in a wide range of institutions [1,2]. Current evidence suggests that PCI can be safely performed in hospitals with low surgical volume and facilities without on-site cardiac surgery units [3,4]. In the 2011 American College of Cardiology Foundation (ACCF)/American Heart Association (AHA)/Society for Cardiovascular Angiography and Interventions (SCAI) guideline, elective PCI was upgraded to Class IIb and primary PCI to Class IIa at facilities without on-site surgery [5]. However, the outcome of PCI procedures may vary in relation to a site’s surgical volume (e.g., volume of coronary artery bypass grafting [CABG]) [6].

The impact of PCI volume in relation to absolute surgical volume has not been studied extensively. Recently, major publications that assessed the PCI volume-outcome relationship at a nationwide level have become available, but these studies have not comprehensively evaluated the association of CABG volume with use and outcome of PCI [7,8]. The present study investigated in-hospital PCI outcome in relation to institutional CABG volume using the nationwide PCI (J-PCI) Registry in collaboration with extracted data from the national Japan Cardiovascular Surgical Database (JCVSD). 

## 2. Methods

### 2.1. Data Sources

#### 2.1.1. Japanese Cardiovascular Intervention and Therapeutics Registry: J-PCI

The J-PCI is a nationwide, prospective, multicenter registry designed to collect clinical data on patients undergoing PCI in Japan, operated by the Japanese Association of Cardiovascular Intervention and Therapeutics. Since January 2013, the J-PCI registry has been incorporated into the National Clinical Database, which is a nationwide prospective web-based registry platform linked to the surgical board certification system. The J-PCI registry is linked to the board certification system in interventional cardiology [9].

The J-PCI continuously communicates with data managers responsible for data collection through the National Clinical Database web-based data management system and performs random site visits to validate the submitted data (20 sites/year). According to the annual reports of the Japanese Registry of All Cardiac and Vascular Disease, 255,416 PCIs were performed during the current study period [10]. Given the total number of 415,025 PCIs registered in J-PCI in 2014 and 2015 (207,512/yr in average), we assume approximately 81.2% coverage of all procedures in Japan in our registry.

#### 2.1.2. Japan Cardiovascular Surgical Database Adult Division: JCVSD Adult Division

The Japan Cardiovascular Surgery Database is a nationwide registry of patients undergoing cardiovascular surgeries in Japan. The data collection is also conducted using the National Clinical Database platform and is linked to cardiovascular surgery certification. Its coverage is assumed to exceed 95% of all major adult cardiovascular surgeries in Japan [11]. It also performs routine on-site audits for data accuracy. The data have been used extensively for clinical and health policy research in the past decade [12].

#### 2.1.3. Study Approval

The present study was approved by the Board of Directors and Scientific Committee of the Japanese Association of Cardiovascular Intervention and Therapeutics, as well as the database committee of the Japan Cardiovascular Surgery Database. The study protocol of the J-PCI registry and its data use for retrospective observational studies was approved by the Institutional Review Board committee at Network for Promotion of Clinical Studies (a specified nonprofit organization affiliated with Osaka University Graduate School of Medicine (Osaka, Japan)) and complied with the Declaration of Helsinki. Similarly, the study protocol of JCVSD and its data use for retrospective observational study was and approved by the ethics committee of the Japan Surgical Society.

### 2.2. Study Patients

**Figure 1:**
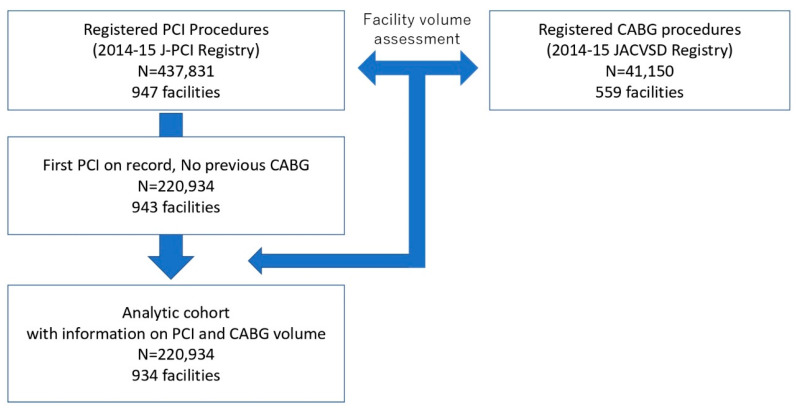
Study analytic flow.

Among all patients in the J-PCI registry, we enrolled those undergoing PCI on native vessels for the first time, aged 20 years or older. Patients without a recorded gender were excluded. Among all patients undergoing CABG in the JCVSD, aged 20 years or older, we enrolled those whose gender was not missing. We ultimately identified 220,934 cases of elective PCI and 41,150 cases of elective CABG performed in 2014–2015 in the two registries (Figure 1). After linking the two datasets using unique identification codes, we identified 934 facilities performing PCI in our analysis cohort.

### 2.3. Hospital Category

We assessed the number of PCI and CABG procedures performed at each hospital during the study period of 2014–2015, and categorized the hospitals into 12 groups by the combination of hospital’s CABG volume (none, 1–49, and ≥50) x PCI volume (1–99, 100–199, 200–399, and ≥400). 

### 2.4. Study Outcomes

The evaluated outcomes included in-hospital death and a composite of periprocedural events including in-hospital death, tamponade (new pericardial effusion which worsens hemodynamics after PCI), shock from heart failure (new episode of systolic blood pressure < 80mmHg, cardiac index < 1.8 L/min/m^2^, and/or the requirement for parenteral inotropic or vasopressor agents or mechanical support to maintain blood pressure and cardiac indexes above those levels after PCI), stent thrombosis (“definite” in a definition of Academic Research Consortium, that is, angiographic or pathological confirmation of stent thrombosis), emergent surgery (urgent or emergent surgery performed during the hospital stay), and post-procedural bleeding (perioperative and/or postoperative bleeding requiring blood transfusion).

### 2.5. Statistical Analysis

We first assessed the baseline characteristics of the patients and PCI procedures according to hospital category based on facilities’ CABG volume and tested for equality in the proportions across groups using Pearson’s chi-squared test. For each hospital category (grouped based on facilities’ PCI and CABG volume as explained in the hospital category section), we calculated the number of hospitals, the number of patients treated, the number of PCI operators, the number of PCIs per operator, and estimated the incidence of outcomes, as well as the predicted probability of mortality for the patients based on their baseline characteristics [13]. We then estimated the odds for in-hospital death and for in-hospital complications for each hospital category relative to the reference group (facilities with 50 or more CABG as well as 400 or more PCIs per year), using a hierarchical logistic regression model including all patient- and procedure-level covariates (listed in Table 1) as fixed effects and hospital-specific random intercepts. 

We repeated the regression analyses in the subgroup of acute coronary syndrome patients. In addition, a sensitivity analysis excluding sites with exceptionally low PCI or CABG numbers. Threshold of <50/yr for PCI, and <15/yr for CABG was chosen for exclusion since prior analysis from our dataset to define PCI institutions that are at higher risk for in-hospital mortality and procedural complications and as an exclusion criterion of volume-outcome analysis for CABG [9,14]. Finally, we repeated the primary analysis using the cut-off values for PCI and CABG volume based on the quartile values of the distribution. All analyses were performed using SAS software version 9.4 (SAS Institute, Cary, NC, USA), and two-tailed *p* values < 0.05 were considered statistically significant.

## 3. Results

Among the 220,934 total cases included in the study cohort, 162,411 were men, with mean age of 69.7 (SD 11.6) years. Patients underwent PCI at hospitals with varying CABG volume. The prevalence of patient comorbidities was similar across groups, with statistically significant but not clinically substantive differences (Table 1). The median number of registered PCI operators generally increased in parallel with the institutional PCI volume. However. the trend was not obvious in high CABG institutions; the median number of PCI per operator was high (~40) even in hospitals with low PCI volume.

The median risk-predicted mortality (adjusted for the variables associated with post-PCI mortality) remained largely equivocal within range of 0.2% to 0.3% and did not differ by the CABG volume or PCI volume. In addition, 95th percentile (percentile range where most of the mortality events occur) was consistently low in high PCI (≥400) category relative to lower PCI volume categories at all CABG volume strata (Table 2A).

## 4. Outcomes

**Figure 2:**
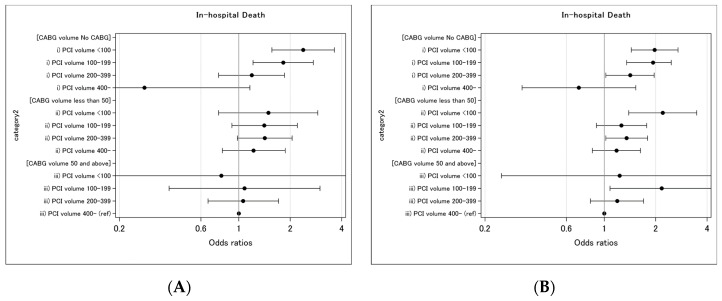
Hazard for in-hospital death (**A**) and composite outcome (**B**) in PCI patients by the volume of CABG. Odds ratios are reported on a log scale.

We observed 2,179 (0.9%) in-hospital deaths and 5,306 (2.4%) composite outcomes. Un adjusted mortality and overall complication rate by absolute volume of the CABG (None, <50, 50 and above) and PCI (<100, <200, <400, and 400 and above) is demonstrated in Table 2A. The crude in-hospital death rate ranged from 0.1 (≥400 PCI—No CABG facilities) to 1.4% (<100 PCI—No CABG facilities), and the complication rate ranged from 0.7 (≥400 PCI—No CABG facilities) to 3.6% (100–399 PCI—≥50 CABG facilities). In hierarchical logistic regression analysis (Table 2B), CABG volume was associated with the in-hospital mortality of PCI at facilities performing less than 200 PCIs per year, but not at facilities performing 200 or more. Similarly, in-hospital mortality or complication was associated with PCI volume <200 only if no CABG is done at the facility, despite similar median risk-predicted mortality in each volume category. A PCI volume-outcome relationship was observed amongst the PCI institutions with no CABG volume, but not within the institutions with CABG volume of ≥50/yr (Figure 2). 

Adjusted in-hospital PCI outcomes for patient subgroup that presented with ACS are demonstrated in Table 3A. In concordance with our main findings, higher risk of in-hospital complications and/or in-hospital death for PCI procedures were observed predominantly in low-PCI (<100 or <200 cases /year) low-CABG (No or less than 50 cases/year) institutions when referenced to high-PCI and CABG institutions. In addition, even after excluding these institutions with extremely low volume of PCI (<50/yr) or CABG (<15/yr), the results remained largely consistent with the main findings (Table 3B). We also performed sensitivity analysis, with both CABG and PCI volumes divided into quartiles (Q1: 0, Q2: 1 to 26. Q3: 27 to 49, and Q4: 50 and above for CABG, and Q1: 1 to 210, Q2: 211 to 313, Q3: 314 to 478, and Q4: 479 and above for PCI) and observed similar trend (Table 4).

## 5. Discussion

This study is the first to assess the effect of risk stratification with institutional volume of PCI in relation to CABG volume on in-hospital outcomes at a nationwide level. Using data from more than 220,000 patients from the J-PCI Registry, we found that in-hospital mortality or complication was associated with PCI volume < 200 only if no CABG is done at the facility. A PCI volume-outcome relationship was observed amongst institutions with no CABG volume, but not among the institutions that performed CABG ≧ 50/year. These results remained largely consistent even after excluding these institutions with extremely low number of PCI or CABG. The institutional CABG volume was extracted from national surgical database (JCVSD), and our study results underscore the importance of seeking an ideal balance between surgical and interventional procedures.

As for the volume-outcome relationship in PCI procedures, Mc Grath et al., in their classic analysis, indicated that increased risk of PCI was primarily confined to low-volume hospitals performing < 50 PCI procedures per year [15]. In addition, Wennberg et al. demonstrated higher risk of adverse outcomes associated with PCI performed at hospitals without on-site cardiac surgery [16]. The reported need for emergency cardiac surgery in the contemporary era of intracoronary stent implantation has lowered to the range of 0.3–0.6% [17,18]. More recent meta-analysis suggested that clinical outcomes and complications after PCI are similar between hospitals with and without on-site surgical backup regardless of the indications for PCI [3]. In addition, a recently published study on this topic pointed out that the volume-outcome relationship might no longer be inverse in the era of radial access and technical iteration of equipment made available to PCI operators. However, our study implies that this result may differ by volume of CABG; the volume–outcome relationship on PCI outcome, although less clear even when a significant number of CABG was performed at the same site, was obvious in the subgroup of institutions that had no surgical back-up. This finding was consistent across subgroups of patients presenting with ACS and excluding institutions with extremely low volume, and in a sensitivity analysis using different volume cutoff. The present analysis also lends support to the hypothesis that patients may have better outcomes at institutions with a higher CABG volume (in reference to PCI volume); PCI at sites with a low ratio (high number of CABG compared to PCI procedures) had consistently lower risk of in-hospital adverse events. Previously, Carley et al demonstrated that the ratio of PCI to CABG was a key determinant of outcomes, beyond the procedure volume [19]. In their study, a statistically significant effect of PCI volume on CABG mortality occurred in lower-volume hospitals when the PCI/CABG ratio exceeds 2.0. This may be related to the reduction in CABG volume and the increase in patient acuity that necessarily follows. Taken together with the present study, it underscores the importance in seeking ideal balance between surgical and interventional revascularization procedures.

There are several possible explanations for our findings. Obviously, within larger hospitals, more experienced PCI operators may be available to either assist on the more difficult cases, provide support when complications occur, or there may be preferential shunting of higher risk cases to more experienced operators within the institution. The 95th percentile value of the predicted risk was consistently low in high PCI volume category (≧400) relative to lower PCI volume categories at all CABG volume strata suggesting patient selection at these facilities. At the same time, it seems that our main result (volume-outcome relationship seen only in no CABG institutions) cannot be explained by ‘selection bias’ alone. If patient selection and associated residual confounding was the sole cause of the observed association between PCI volume and outcome, we would have seen similar association among the CABG performing (both <50 and ≥50) hospital groups.

The presence of experienced surgeons, rather than mere surgical back-up, may be needed for appropriate patient referral and lead to the ideal application of individual revascularization techniques. Successful implementation of the Heart Team in routine care has great potential to positively influence the quality of cardiovascular care [5]. The Heart Team serves to integrate the input of cardiovascular specialists, which aids in minimizing fragmented communication between specialists and eliminates specialty bias in the decision-making process [20]. Interestingly, when the patient’s characteristics were compared between the patients who underwent PCI in the no or low vs. high CABG hospitals, there were significantly more patients with older age and those presenting with unstable angina and acute heart failure (despite lower number of emergent procedures). This indicates that the semi-urgent cases (e.g., patients with unstable angina and/or acute heart failure) and stable but complex PCI cases seems to be the dominant in institutions with no or lower volume surgical back-up. Although these cases do not require PCI emergently, they typically need precise risk stratification and detailed Heart Team discussion [21]. Numerous studies have indicated that precise risk assessment is cardinal in pre-procedural assessment of elective but complex PCI and this might be particularly important in no or lower volume CABG hospitals [13,22,23]. Further, the application of appropriateness use criteria for PCI [24] to real-world clinical practice has demonstrated that the rates of inappropriate PCI procedures vary markedly across hospitals, which suggests the strong possibility of improving PCI outcome from ‘balanced-approach’, represented by ideal surgical and interventional balance [24,25,26]. 

A high-volume surgical back-up team would also likely possess ideal surgical skills for ‘bailing-out’ from interventional emergencies. This concept seems still valid after advancement in PCI technology, technique, adjunctive pharmacotherapy, and operator experience have substantially lowered the rates of complications requiring emergency cardiac surgery. Hollingsworth et al. demonstrated that health systems with physicians who tend to work together in tightly-knit groups during CABG episodes realize better surgical outcomes [27]. Consequently, PCI sites that focus on teamwork between medical and surgical care providers may have positive effects on their care.

While the high number of PCI volume may reflect a variety of factors, for example, local style of practice and favorable reimbursement policies as well as physician and patient belief in the benefits of PCI, those factors do not explain why PCI-dominant sites with no or a few CABG volume had higher risk of adverse events. This finding could be expected, given that undergoing elective PCI does not appear to improve mortality for patients with stable coronary artery disease [28], and may indicate that an experienced surgical team is particularly important for deciding in which patients the benefits of PCI outweigh potential harms, particularly in light of the efficacy of optimal medical therapy. Our results also indicated that low number of PCI procedures performed by individual PCI operator may partially account for the higher risk of adverse events in facilities with no or low CABG. Regardless of reason, our results suggest that efforts to appropriately balance the revascularization procedures should be encouraged.

## 6. Limitations

Our findings should be interpreted in the context of several potential limitations. First, this is a retrospective cohort study using data from clinical registries. As allocations of PCI cases to the hospital groups are not randomized, our estimates are vulnerable to potential confounding by unmeasured risk factors. However, the selected variables included in the J-PCI are the predictors for in-hospital mortality detected in the development of the National Cardiovascular Data Registry Cath PCI Risk Score System [13], suggesting an adequate adjustment for patient case-mix in our analysis. Second, in-hospital death may be underreported at sites without surgical back-up, which account for roughly half of the participating institutes in J-PCI, as a post-procedural transfer to an outside surgical center is coded as an “emergent surgery”, a component of secondary outcome, regardless of subsequent outcome after the surgery. However, since we could capture such an event as a secondary outcome and the volume-outcome relationships for primary and secondary outcomes demonstrated similar trends in this study, we believe our findings are robust even if in-hospital deaths were underestimated. Third, in Japan, a large number of PCI institutes have been developed, and in our dataset, almost half of the participating institutions did not reach the ACCF/AHA/SCAI target of more than 200 PCIs per year per institute, which may affect the generalizability of our findings to other settings where these criteria are strictly enforced. Finally, this study focused on in-hospital outcomes. Lastly, our studied registries did not allow linkage of patients across facilities, and we were not able to identify patients referred for CABG outside of the PCI facility. However, the number of cases that required emergency surgery was no more than 0.12%, and when restricted to procedures from no-CABG hospitals, the percentage was even lower at 0.10%. Therefore, we estimate that the number of referrals within no-CABG hospitals was relatively small and would have minimum impact on our main results.

## 7. Conclusions

In this collaborative analysis of PCI and CABG nationwide registries in Japan, facilities’ CABG volume was associated with patients’ clinical outcome after PCI, when the volume of PCIs performed at the facility was limited. Seeking ideal balance between surgical and interventional revascularization procedures may result in improved outcomes.

## Figures and Tables

**Table 1:** Patient characteristics of percutaneous coronary intervention (PCI) patients by the absolute volume of coronary artery bypass grafting (CABG).

CABG Volume Category	No CABG	1–49	50 and above	*p*-Value
N (total = 220,934)	56,580	110,459	53,895	
**Age category**				<0.001
20–39	391 (0.7%)	1046 (1.0%)	450 (0.8%)	
40–59	9058 (16.0%)	19,529 (17.7%)	9498 (17.6%)	
50–74	24,885 (44.0%)	49,934 (45.2%)	24,677 (45.8%)	
75 and above	22,246 (39.3%)	39,950 (36.2%)	19,270 (35.8%)	
**Diagnosis**				<0.001
Stable Angina	17,688 (31.3%)	33,706 (30.5%)	18,654 (34.6%)	
Unstable Angina	10,663 (18.9%)	18,332 (16.6%)	8648 (16.1%)	
Acute Myocardial Infarction	17,927 (31.7%)	33,706 (30.5%)	18,654 (34.6%)	
Old Myocardial Infarction	1572 (2.8%)	3095 (2.8%)	1494 (2.8%)	
Silent Myocardial Ischemia	8267 (14.6%)	12,062 (10.9%)	6030 (11.2%)	
Other	463 (0.8%)	1070 (1.0%)	430 (0.8%)	
**Emergent procedure**	21,140 (37.4%)	49,042 (44.4%)	21,981 (40.8%)	<0.001
**Number of Vessels treated**				<0.001
1	51,221 (90.5%)	98,451 (89.1%)	47,880 (88.8%)	
2	3127 (5.5%)	6398 (5.8%)	3285 (6.1%)	
3 or include LMT	2232 (3.9%)	510 (5.1%)	2730 (5.1%)	
**Preprocedural condition**				
Cardiac Shock within 24 h of procedure	2093 (3.7%)	5347 (4.8%)	1979 (3.7%)	<0.001
Acute heart failure	3032 (5.4%)	6419 (5.8%)	2637 (4.9%)	<0.001
Cardiac Arrest within 24 h	904 (1.6%)	2933 (2.7%)	1195 (2.2%)	<0.001
**Comorbidities**				
Past Heart Failure	4830 (8.5%)	9647 (8.7%)	4905 (9.1%)	<0.001
COPD	969 (1.7%)	1960 (1.8%)	947 (1.8%)	0.66
Hemodialysis	2227 (3.9%)	4715 (4.3%)	2562 (4.8%)	<0.001
Renal dysfunction	6633 (11.7%)	15,322 (13.9%)	7591 (14.1%)	<0.001
Diabetes	21,395 (37.8%)	41,911 (37.9%)	20,167 (37.4%)	0.12
AAA/PAD	2946 (5.2%)	5813 (5.3%)	3258 (6.1%)	<0.001
Hypertension	39,412 (69.7%)	77,633 (70.3%)	39,180 (72.7%)	<0.001
**Number of facilities**	437	389	117	
**Mean annual Case Volume**				<0.001
1–99	197 (45.1%)	35 (9.0%)	3 (2.6%)	
100–199	145 (33.2%)	83 (21.3%)	9 (7.7%)	
200–399	87 (19.9%)	194 (50.1%)	53 (45.3%)	
400 and above	8 (1.8%)	76 (19.5%)	52 (44.4%)	
**Outcomes**				
Deaths	535 (1.0%)	1221 (1.1%)	423 (0.8%)	<0.001
Composite Outcome	1426 (2.5%)	2831 (2.6%)	1049 (2.0%)	<0.001

**Table 2:** Crude (**A**) and adjusted (**B**) in-hospital PCI outcomes by its volume; subcategorized by absolute CABG volume (none, less than 50, and 50 and above/year).

**A.**				
**Yearly CABG Volume**	**Yearly PCI Volume**			
	**1–99**	**100–199**	**200–399**	**400 and above**
**None**				
Number of Facilities	200	145	87	8
Number of Patients	10,429	20,680	21,390	4081
In-Hospital Death	144 (1.4%)	231 (1.1%)	157 (0.7%)	3 (0.1%)
In-Hospital Complications	315 (3.0%)	601 (2.9%)	482 (2.3%)	28 (0.7%)
Number of Registered PCI Operators, median (min-max)	2 (1–11)	4 (1–13)	6 (1–12)	8.5 (1–14)
Number of PCI per operator	14	25	35	50.5
Predicted mortality, median (p5–p95)	0.2% (0.04–2.7)	0.2% (0.04–2.9)	0.2% (0.04–2.6)	0.1% (0.04–1.5)
Ratio (PCI/CABG) median (p25–p75)	–	–	–	–
**1–49**				
Number of Facilities	36	83	195	76
Number of Patients	2442	13,600	56,666	37,751
In-Hospital Death	25 (1.0%)	148 (1.1%)	663 (1.2%)	385 (1.0%)
In-Hospital Complications	85 (3.5%)	338 (2.5%)	1518 (2.7%)	890 (2.4%)
Number of Registered PCI Operators, median (min-max)	3 (1–10)	5 (1–24)	8 (1–20)	10 (4–23)
Number of PCI per operator	11	20	27	39
Predicted mortality, median (p5–p95)	0.2% (0.04–4.3)	0.3% (0.04–3.8)	0.2% (0.04–4.3)	0.2% (0.04–2.8)
Ratio (PCI/CABG) median (p25–p75)	5.2 (2.5–9.2)	7.9 (5.0–11.6)	11.4 (8.2–15.8)	17.9 (12.8–26.7)
**50 and above**				
Number of Facilities	3	9	53	52
Number of Patients	194	1291	16,285	36,125
In-Hospital Death	2 (1.0%)	12 (0.9%)	154 (1.0%)	255 (0.7%)
In-Hospital Complications	4 (2.1%)	47 (3.6%)	382 (2.4%)	616 (1.7%)
Number of Registered PCI Operators, median (min-max)	1 (1–5)	6 (2–24)	10 (4–28)	15 (6–36)
Number of PCI per operator	40	10.5	19	32
Predicted mortality, median (p5–p95)	0.3% (0.04–12.1)	0.2% (0.04–4.0)	0.2% (0.04–3.5)	0.2% (0.04–2.4)
Ratio (PCI/CABG) median (p25–p75)	0.5 (0.01–1.7)	2.0 (1.5–2.7)	4.2 (2.9–5.1)	7.5 (5.1–10.5)
**B.**				
**Yearly CABG Volume**	**Yearly PCI Volume**			
	**1–99**	**100–199**	**200–399**	**400 and above**
**None**				
OR for in-hospital death	2.38 (1.56–3.63)	1.82 (1.21–3.73)	1.19 (0.76–1.85)	0.28 (0.07–1.16)
OR for in-hospital complications	1.97 (1.44–2.70)	1.83 (1.35–2.47)	1.42 (1.02–1.96)	0.71 (0.33–1.53)
**1–49**				
OR for in-hospital death	1.49 (0.76–2.90)	1.41 (0.91–2.20)	1.42 (0.98–2.05)	1.22 (0.80–1.87)
OR for in-hospital complications	2.20 (1.39–3.48)	1.26 (0.90–1.77)	1.35 (1.02–1.79)	1.18 (0.85–1.63)
**50 and above**				
OR for in-hospital death	0.79 (0.08–7.86)	1.08 (0.39–2.99)	1.06 (0.66–1.71)	reference group
OR for in-hospital complications	1.23 (0.25–5.91)	2.17 (1.08–4.36)	1.19 (0.83–1.70)	reference group

Abbreviation: CABG coronary artery bypass graft, PCI percutaneous coronary intervention, OR odds ratio.

**Table 3:** Adjusted in-hospital PCI outcomes by its volume for patient subgroup that presented with acute coronary syndrome (ACS) (**A**) or with exclusion of institutions with extremely low volume of PCI (<50 cases/yr) or CABG (<15 cases/yr) (**B**) subcategorized by absolute CABG volume (none, less than 50, 50 or above).

(A) With inclusion of ACS patients only.				
**Yearly CABG Volume**	**Yearly PCI Volume**			
	**1–99**	**100–199**	**200–399**	**400 and above**
**None**				
OR for in-hospital death	2.45 (1.58–3.78)	1.82 (1.20–2.76)	1.28 (0.81–2.03)	0.34 (0.08–1.46)
OR for in-hospital complications	2.09 (1.47–2.97)	2.00 (1.43–2.80)	1.65 (1.14–2.37)	0.85 (0.36–2.03)
**1–49**				
OR for in-hospital death	1.56 (0.79–3.07)	1.39 (0.88–2.2)	1.42 (0.97–2.08)	1.18 (0.76–1.82)
OR for in-hospital complications	2.33 (1.40–3.87)	1.31 (0.90–1.90)	1.45 (1.06–1.99)	1.21 (0.85–1.74)
**50 or above**				
OR for in-hospital death	0.80 (0.08–8.20)	0.99 (0.34–2.89)	1.01 (0.62–1.66)	reference group
OR for in-hospital complications	1.68 (0.31–8.95)	2.04 (0.93–4.45)	1.20 (0.81–1.79)	reference group
(B) With exclusion of institutions with extremely low number of PCI (<50/yr) or CABG (<15/yr) volume.				
**Yearly CABG Volume**	**Yearly PCI Volume**			
	**50–99**	**100–199**	**200–399**	**400 and above**
**None**				
OR for in-hospital death	2.43 (1.54–3.84)	1.82 (1.21–2.74)	1.19 (0.76–1.86)	0.28 (0.07–1.17)
OR for in-hospital complications	2.13 (1.53–2.96)	1.83 (1.36–2.47)	1.42 (1.03–1.95)	0.71 (0.33–1.51)
**15–49**				
OR for in-hospital death	0.88 (0.31–2.56)	1.29 (0.79–2.10)	1.39 (0.95–2.05)	1.29 (0.83–2.00)
OR for in-hospital complications	1.33 (0.66–2.68)	1.23 (0.86–1.77)	1.29 (0.97–1.71)	1.22 (0.88–1.70)
**50 or above**				
OR for in-hospital death	1.03 (0.08–12.95)	1.08 (0.39–3.00)	1.06 (0.66–1.71)	reference group
OR for in-hospital complications	0.44 (0.05–3.88)	2.17 (1.09–4.31)	1.19 (0.84–1.70)	reference group

Abbreviation: ACS acute coronary syndrome, CABG coronary artery bypass graft, PCI percutaneous coronary intervention, OR odds ratio.

**Table 4:** Adjusted in-hospital PCI patient risk by its volume; subcategorized by CABG volume in quartiles.

Yearly CABG Volume	Yearly PCI Volume			
	Max 210	Max 313.5	Max 478.5	Above 478.5
**None**				
OR for in-hospital death	2.02 (1.34–3.05)	1.12 (0.67–1.87)	0.91 (0.37–2.21)	0.13 (0.01–1.35)
OR for in-hospital complications	1.82 (1.34–2.47)	1.39 (0.96–2.02)	0.86 (0.46–1.63)	0.71 (0.27–1.84)
**Max 26.5**				
OR for in-hospital death	1.73 (1.08–2.78)	1.50 (0.92–2.43)	1.15 (0.68–1.94)	0.98 (0.46–2.07)
OR for in-hospital complications	1.62 (1.14–2.31)	1.26 (0.88–1.82)	1.09 (0.73–1.61)	0.7 (0.39–1.24)
**Max 49**				
OR for in-hospital death	1.05 (0.58–1.90)	1.32 (0.8–2.18)	1.23 (0.75–2.03)	1.56 (0.86–2.85)
OR for in-hospital complications	1.23 (0.80–1.91)	1.28 (0.88–1.88)	1.13 (0.77–1.65)	1.68 (1.06–2.65)
**50 and above**				
OR for in-hospital death	1.20 (0.52–2.77)	1.05 (0.58–1.91)	1.09 (0.62–1.90)	reference group
OR for in-hospital complications	2.01 (1.12–3.62)	1.16 (0.75–1.81)	1.01 (0.66–1.55)	reference group

Abbreviation: CABG: coronary artery bypass graft, PCI: percutaneous coronary intervention, OR: odds ratio.

## List of Abbreviations

ACCF	American College of Cardiology Foundation
AHA	American Heart Association
CABG	coronary artery bypass grafting
JCVSD	Japan Cardiovascular Surgical Database
J-PCI	PCI Registry for Japanese Association of Cardiovascular Intervention & Therapeutics
PCI	percutaneous coronary intervention
SCAI	Society for Cardiovascular Angiography and Interventions
